# Detection of MRI-Invisible Disease Using PSMA PET/CT in a Patient Considering Focal Therapy

**DOI:** 10.1155/criu/2981515

**Published:** 2025-03-26

**Authors:** Jae Woong Jang, Aaron Abrams, Anugayathri Jawahar, Hatice Savas, Ximing J. Yang, Vikas Mehta, Marina Schnauss, Edward M. Schaeffer, Ridwan Alam, Ashley E. Ross

**Affiliations:** ^1^Department of Urology, Northwestern University Feinberg School of Medicine, Chicago, Illinois, USA; ^2^Department of Radiology, Northwestern University Feinberg School of Medicine, Chicago, Illinois, USA; ^3^Department of Pathology, Northwestern University Feinberg School of Medicine, Chicago, Illinois, USA

**Keywords:** focal therapy, MRI invisible, PSMA PET/CT, transperineal biopsy

## Abstract

Patient selection for focal therapy (FT) of prostate cancer requires the assessment of MRI and biopsy results. However, there is currently little guidance for the utility of PSMA PET/CT in FT planning. We describe the case of a man originally considered an ideal candidate for FT based on biopsy and MRI who was found to have a contralateral lesion-harboring cancer detected only on PSMA PET/CT.

**Trial Registration:** ClinicalTrials.gov identifier: NCT05852041

## 1. Introduction

In patients with clinically localized prostate cancer, focal therapy (FT) can selectively treat cancerous tissue while minimizing the side effects often associated with whole-gland treatment. The success of FT depends on correct disease localization. Widespread availability and adoption of prostate magnetic resonance imaging (MRI) has helped facilitate the emergence of FT. Currently, ideal candidates for FT, per the FocAL therapy CONsensus (FALCON) project, are individuals with MRI-visible, intermediate-grade, localized prostate cancer [1]. While the panel recognized that clinically significant prostate cancer (csPCa) can be MRI-invisible, there was no consensus as to the use of PSMA PET/CT as an adjunct to MRI for patient selection or in follow-up.

Here, we describe a case favorable for FT based on multiparametric MRI (mpMRI) and biopsy subsequently found to have clinically significant, contralateral prostate cancer based on PSMA PET/CT targeting of an MRI-invisible lesion.

## 2. Case Presentation

**Figure 1:**
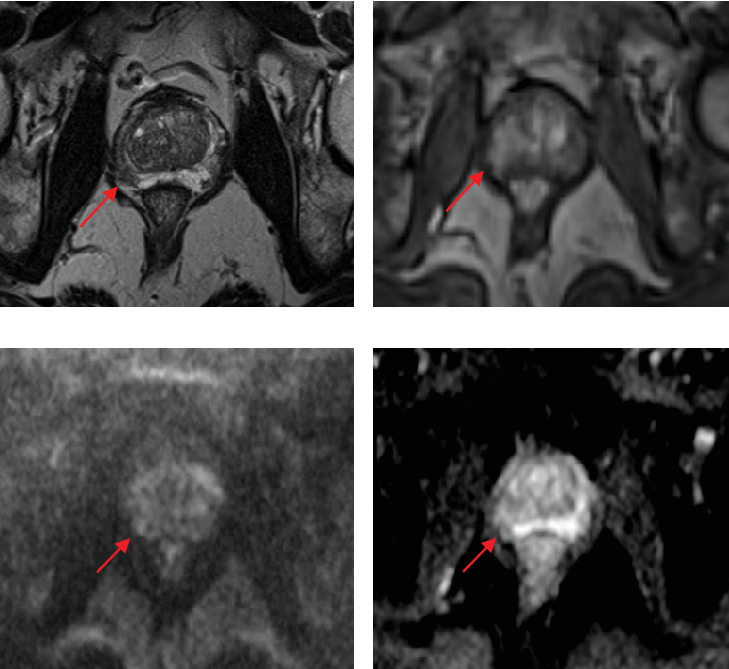
Representative MRI of the right base posterior peripheral zone lesion measuring 0.6 × 0.4 cm (red arrows). This lesion was assigned a PI-RADS 3 score. (a) T2-weighted, (b) postcontrast T1-weighted, (c) diffusion-weighted imaging (DWI), and (d) apparent diffusion coefficient (ADC).

**Figure 2:**
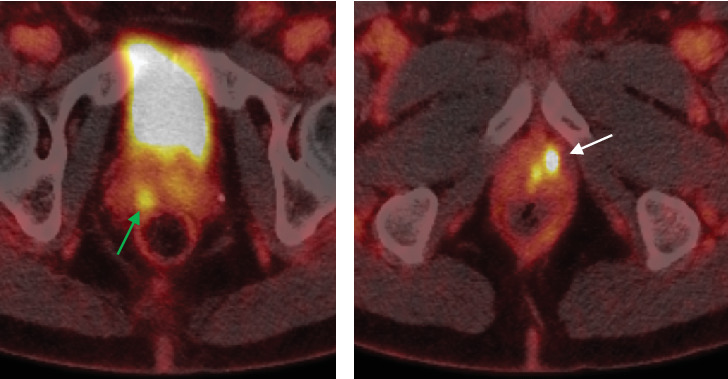
Representative Piflufolastat F-18 PSMA-PET/CT images revealing two PET-avid lesions: (a) right base posterior lesion (green arrow) and (b) left apex anterior lesion (white arrow).

A 65-year-old man with a family history of localized prostate cancer and personal history of deep venous thrombosis and appendectomy was referred to his local urologist for work-up of an elevated PSA. His PSA rose from 3.7 ng/mL in October 2020 to 8.2 ng/mL in November 2023, triggering mpMRI and prostate biopsy. MRI demonstrated a 34-cc gland (PSA density of 0.24 ng/mL/cc) and a 0.6 × 0.4 cm lesion at the posterolateral peripheral zone of the right base, assigned a PI-RADS 3 score (Figure 1). Transrectal (TR) 12-core prostate biopsy at an outside institution demonstrated Gleason Grade Group (GG) 3 disease in the right base, involving 20% of one core. Given his unfavorable, intermediate risk disease, he was staged with a Piflufolastat F-18 PSMA PET/CT scan, which showed no evidence of metastatic disease but revealed two foci of increased radiotracer activity within the prostate. One focus was located in the medial right base with a maximum standardized uptake value (SUVmax) of 4.0. The other focus of PET activity was located in the left anterior apex—an area where biopsy did not detect cancer and MRI showed no lesion—with an SUVmax of 15.0 (Figure 2). Internal review of the biopsy slides resulted in downgrading of the singular positive core from GG 3 to GG 2, involving 20% of the core with 20% Gleason Pattern 4. This core was sent for Decipher Genomic Analysis (Veracyte, Inc., San Francisco, CA) and was classified as low risk (Decipher Score 0.28). Options were discussed, and the decision to perform a transperineal (TP) biopsy of the PET-avid, MRI-invisible left anterior lesion was made.

**Figure 3:**
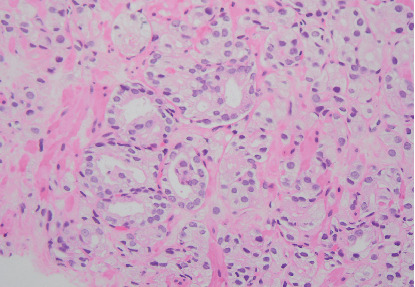
Representative histopathologic image from targeted transperineal biopsy of the PET-avid left anterior lesion. The final diagnosis of this lesion was GG2 adenocarcinoma with 40% Gleason Pattern 4.

Final pathology from this area found GG 2 disease with 40% Gleason Pattern 4 (Figure 3). A Decipher test from this specimen revealed intermediate risk (Decipher Score 0.46).

**Figure 4:**
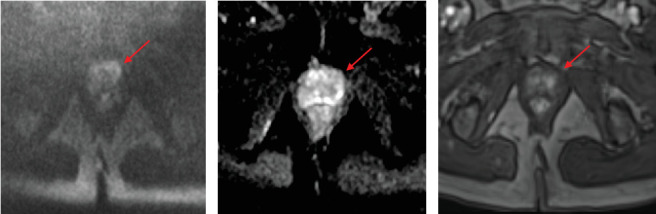
Representative MRI of left apex anterior lesion (red arrows). (a) DWI, (b) ADC, and (c) dynamic postcontrast. There is a subtle, asymmetric, increased signal noted retrospectively on DWI that corresponds to the lesion demonstrating hypermetabolic activity on PSMA PET/CT. There is a very mild hypointense signal on ADC with asymmetric early enhancement on postcontrast images. This lesion was assigned a PI-RADS score of 4.

Upon repeat review of the initial prostate MRI, a subtle lesion of PI-RADS score 4 was noted in the left anterior apex (Figure 4). However, due to the location and minuscule size of the lesion, it was reported that this lesion would have been unlikely to be detected without the knowledge gained from the PSMA PET/CT. Though the patient had initially favored FT, given the current results of bilateral intermediate risk disease, whole-gland treatment is now being pursued.

This case report follows the principles of the Declaration of Helsinki. Informed consent for de-identified use of patient information was obtained under an IRB-approved protocol.

## 3. Discussion

We present the case of a patient who was thought to be an ideal candidate for FT based on an MRI-visible lesion but was ultimately found to have more extensive disease detected after PSMA PET/CT. Embedded within this case are several timely topics for discussion.

Patient outcomes following FT hinge on the accurate assessment, diagnosis, and mapping of prostate cancer. Although an MRI is considered standard prior to consideration of FT, 10%–30% of csPCa can be MRI invisible [2–4]. While many of these cases are detected through systematic biopsies, some lesions—particularly those in the anterior prostate—may be under-sampled by TR biopsy, as in this case. TP biopsy has had increased adoption in urology practices as it allows for reduced infection rates without the need for periprocedural antibiotics [5]. In addition, studies have reported that TP biopsy can outperform TR biopsy in detecting csPCa of the anterior and apical prostate, with a recent meta-analysis of predominantly retrospective studies involving over 8000 patients reporting that MRI-guided TP biopsies had 2.2 and 1.9 times higher odds of detecting anterior and apical disease, respectively, when compared to MRI-guided TR biopsies [6]. Evidence is conflicting, however, with the PERFECT randomized trial, published after the meta-analysis, failing to find a difference between TP and TR biopsy in the detection of either apical or anterior lesions [7]. The ongoing TRANSLATE randomized clinical trial similarly aims to determine whether MRI-informed TP biopsy improves the detection of csPCa compared to MRI-informed TR biopsy and may add clarity to whether TP biopsies truly have an advantage in detecting apical and anterior cancers when compared to TR biopsies [8].

The role of PSMA PET/CT in determining FT candidacy is unclear. In this case, the patient was considered an optimal candidate for FT prior to the PSMA PET/CT—he had a single positive core on biopsy that corresponded well to the MRI-visible lesion. However, the PSMA PET/CT changed the trajectory of this patient's care, revealing an MRI-invisible but PET-avid contralateral lesion that was confirmed to harbor csPCa. Current consensus on FT including the FALCON project recommends against the use of PSMA PET/CT as a replacement for MRI in patient selection, but they do not offer guidance on the utility of using PSMA PET/CT as an adjunct to MRI [1]. The prospective, multicenter PRIMARY trial originating from Australia found that the combination of PSMA PET/CT with MRI reduced the number of false negatives in detecting csPCa when compared to MRI alone [9]. This suggests a potential benefit in adding PSMA PET/CT to MRI, but the study did not investigate the differences in each modality to identify individual prostate lesions. Nevertheless, there is clearly a proportion of patients who may benefit from additional PSMA-based imaging to detect occult disease. Along these lines, our group is currently evaluating the ability of 18F-radiohybrid (rh) PSMA-7.3 PET/MRI to detect higher risk disease among patients with low or favorable intermediate risk prostate cancer and Decipher Score ≥ 0.45. Findings from this study may allow for additional risk stratification of patients considering FT and/or provide greater confidence in treatment with FT.

In conclusion, we present the case of a patient who was thought to be an ideal candidate for FT based on MRI and TR biopsy results but was found to have more extensive disease that was detected after PSMA PET/CT. The subsequent change in management highlights a potential role for PSMA PET/CT in qualifying candidates for FT.

## Data Availability

Data sharing is not applicable to this article as no new data were created or analyzed in this study.
